# Outcomes of referrals from endodontic to orofacial pain specialists: A retrospective cohort study

**DOI:** 10.1002/cre2.497

**Published:** 2021-10-08

**Authors:** Ozge Erdogan, Austin Ramsey, James M. Uyanik, Jennifer L. Gibbs, Lorel E. Burns

**Affiliations:** ^1^ Department of Endodontics, College of Dentistry New York University New York New York USA; ^2^ Division of Endodontics in Restorative Dentistry and Biomaterials Sciences Harvard School of Dental Medicine Boston Massachusetts USA; ^3^ Department of Oral and Maxillofacial Pathology, Radiology and Medicine College of Dentistry, New York University New York New York USA

**Keywords:** endodontics, orofacial pain, root canal treatment, TMD

## Abstract

**Objectives:**

Diagnosis and treatment of non‐odontogenic pain is challenging for endodontists. The purpose of the study was to investigate the outcomes of referrals to orofacial pain specialists made for patients with suspected non‐odontogenic pain, after evaluation and/or treatment by an endodontist.

**Materials and Methods:**

A retrospective review of dental records was conducted for 60 patients referred from a postgraduate endodontic clinic to an orofacial pain clinic. Patient demographics, pain history, endodontic, and orofacial pain diagnoses were collected. Number of visits, length of treatment, and treatments prescribed were recorded. For analysis of outcomes, data pertinent to resolution/persistence of symptoms and patient compliance were analyzed.

**Results:**

Thirty‐five patients were included in the study. The most frequent pulpal and periapical diagnoses were previously treated (62%) and symptomatic apical periodontitis (72%), respectively. The most common orofacial pain diagnosis was temporomandibular disorder. The average time spent to diagnose and treat the pain was 17 months. Pain reduction varied and was documented for 51% of patients. Indications of non‐compliance with orofacial pain appointments and treatments were documented for 66% of patients.

**Conclusions:**

Non‐odontogenic pain diagnosis and treatment are challenging. Patients may have an increased predilection for developing persistent pain after endodontic treatment and/or have an undiagnosed, chronic orofacial pain condition as a true source of their chief complaint. It may be helpful for endodontists to set expectations of typical treatment times/plans when referring patients for evaluation and treatment of non‐odontogenic pain.

## 1 INTRODUCTION

Toothache is one of the most prevalent types of orofacial pain reported in the United States (Lipton et al., 1993). In some cases, the diagnosis of dental pain can become quite difficult. Pain, seemingly originating from teeth, may in fact have a non‐odontogenic source and be referred to the teeth from other structures such as muscles, joints, the maxillary sinus, and adjacent cranial nerves (e.g., trigeminal neuralgia). To further complicate diagnostic efforts, non‐odontogenic orofacial pain can occur concurrently with true odontogenic pain (Benoliel et al., 2008; Linn et al., 2007; Wright, 2000; Wright & Gullickson, 1996).

In addition to common, chronic orofacial pain conditions such as temporomandibular disorders (TMD), headaches, and trigeminal neuralgia, it is now well recognized that pain can persist after invasive dental treatment, including after endodontic treatment. (Nixdorf et al., 2010; Philpott et al., 2019; Polycarpou et al., 2005). Orofacial pain of non‐odontogenic origin can be a risk factor for the persistence of pain after successful endodontic treatment (Philpott et al., 2019; Polycarpou et al., 2005). Persistent pain is estimated to affect 5%–24% of endodontic patients, and it is not always clear whether such pain is a true sequela of endodontic treatment, or if it is a persistence of pre‐operative pain that was not resolved with root canal treatment (Macrae, 2008; Nixdorf et al., 2010; Philpott et al., 2019; Polycarpou et al., 2005; Vena et al., 2014). Diagnosing such pain is difficult (Aggarwal et al., 2008; Devine et al., 2017, 2018). According to the most recent orofacial pain classification, pain caused by an identifiable trauma to the trigeminal nerve, persisting longer than 3 months, and associated with somatosensory changes is diagnosed as post‐traumatic trigeminal neuropathic pain (PTTNP). Whereas pain, in the absence of a preceding causative event, localized to dentoalveolar site and lasting longer than 3 months is described as persistent idiopathic dentoalveolar pain (PIDAP) (ICOP, 2020). Summarily, differentiating between odontogenic pain caused by active endodontic disease and chronic non‐odontogenic orofacial pain disorders, including persistent pain after endodontic treatment is very challenging for clinicians.

When the source of a patient's chief complaint is suspected to be of non‐odontogenic origin, he/she may be referred to an orofacial pain specialist for further evaluation and treatment. Previous studies have investigated patients referred to orofacial pain specialists in order to evaluate referral patterns, diagnoses, treatments and frequencies of pain resolution (Beecroft et al., 2013; Dieb et al., 2017; Lang et al., 2016; Linn et al., 2007). However, to our knowledge, there are no studies that have evaluated the outcomes of referrals specifically from endodontists to orofacial pain specialists.

*This study aims to*:

1. report patient demographics, diagnostic outcomes and treatments resulting from endodontists' referrals to orofacial pain specialists;
2. describe the treatment timeline for patients referred to an orofacial pain specialist; and
3. measure the incidence of pain relief in patients referred from endodontists to orofacial pain specialists.

## 2 MATERIALS AND METHODS

A retrospective cohort study was conducted as a review of electronic dental records, using Axium Database (Exan, British Columbia). The study and our application for waiver of authorization and/or consent was approved by the Institutional Review Board of the New York University School of Medicine (study number: i18‐01448). All patients that were referred from the New York University College of Dentistry (NYUCD) Post‐Graduate (PG) Endodontic Clinic to the Orofacial Pain Clinic (OFPC) between January 2015 and August 2018 were evaluated for inclusion. The start date of January 2015 was selected because it was the date electronic records were implemented for use at NYUCD.

### 2.1 Inclusion/exclusion criteria

Sixty charts were reviewed. The study inclusion criteria required patients to have attended at least one visit to the OFPC, after being referred from the PG Endodontic clinic. Subjects were excluded if they had inaccessible or incomplete patient records.

### 2.2 Data collection

Electronic dental records were reviewed for each subject. For included subjects, data was collected on patient demographics, medical history, endodontic and orofacial pain diagnoses, treatment times, and outcomes. Collected patient demographics and medical history included age, gender, history of reported chronic pain, and reported depression and/or anxiety. Endodontic and orofacial pain diagnoses were collected from treatment notes. Endodontic diagnoses were made in accordance with the American Association of Endodontists diagnostic guidelines. The number of visits, length of treatment (in months), medications and treatments prescribed were recorded. For analysis of treatment outcomes, data pertinent to the resolution or persistence of clinical symptoms, patient treatment trajectories and patient compliance were analyzed. Pain resolution was captured as change in pain intensity on numerical rating scale relative to the pain intensity reported at the subjects' initial visit.

### 2.3 Statistical analysis

Study data were collected and managed using REDCap electronic data capture tools hosted by New York University Langone Medical Center (Harris et al., 2009). Data were exported from RedCap to Excel (Microsoft, Seattle, WA, United States), and then imported into StataVersion 16 (StataCorp, College Station, TX, United States). Descriptive analysis of variables was performed to identify frequencies, means, and standard deviations.

## 3 RESULTS

### 3.1 Patient demographics and medical history

**Figure 1:**
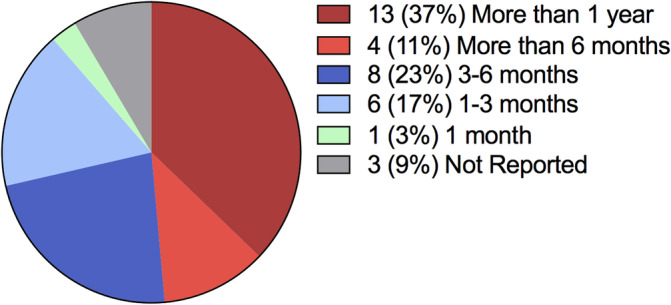
Reported duration of pain related to the chief complaint at the initial orofacial pain visit

Thirty‐five subjects were included in the study. Out of the 35 study subjects, 28 (80%) were female and the mean age was 48 ± 17.4. Forty‐three percent (15/35) self‐reported a history of depression/anxiety. Sixty percent (21/35) reported a history of a pre‐existing chronic pain disorder, distinct from their chief orofacial pain complaint (Table 1). At the first orofacial pain appointment, 13 (37%) reported having endured the pain from their reported chief complaint for more than 1 year, 12 (34%) reported having pain more than 3 months and less than 1 year (Figure 1).

**Table 1:** Patient‐related characteristics and treatment timelines

Age (years), mean ± standard deviation, (range)	48 ± 17.4 (18–88)
Sex, *n* (%)	
Female	28 (80)
Male	7 (20)
Depression/anxiety *n* (%)	15 (43)
History of chronic pain diseases *n* (%)	21 (60)
Arthritis	7 (33)
Headaches/migraines	7 (33)
Sinusitis	3 (14)
Fibromyalgia	1 (5)
Neuropathic pain	1 (5)
Trigeminal neuralgia	1 (5)
TMD	1 (5)
Number of endodontic treatment visits mean ± standard deviation, (range)	3.7 ± 2.0 (2–10)
Number of OFP visits mean ± standard deviation, (range)	2.8 ± 2.4 (1–13)
Number of patients visited dental emergency care *n* (%)	21 (60)
Mean wait time for OFP appointment in days mean ± standard deviation, (range)	72 ± 81.0 (0–398)
Total duration of care, in months mean ± standard deviation (range)	17 ± 12.6 (1–41)

*Note*: *n* (%): number of patients (percentages). Total number of subjects = 35.

Abbreviations: OFP, orofacial pain; TMD, temporomandibular disorder.

### 3.2 Endodontic diagnosis, treatment plans

After initial endodontic consultation, 13 patients (37%), were not treatment planned for endodontic therapy because their pain was suspected to be of non‐odontogenic origin. However, the remaining 22 (63%) patients were treatment planned for endodontic treatment on one or multiple teeth (Table 2). The diagnoses of teeth planned for endodontic treatment are listed in Table 2. The most frequently reported pulpal diagnosis as previously treated (62%), the most frequently reported periapical diagnosis was symptomatic apical periodontitis (72%).

**Table 2:** Endodontic treatment related characteristics

Type of imaging modality *n* (%)	
Radiographs	35 (100)
CBCT	6 (17)
History of endodontic treatment in the affected quadrant *n* (%)	25 (71)

Endodontic treatment plan	Number of patients (%)
No endodontic treatment planned	13 (37)
Primary non‐surgical root canal treatment	11 (31)
Non‐surgical retreatment	9 (27)
Surgical retreatment	1 (3)
Non‐surgical and surgical retreatment Surgical endodontic treatment	1 (3)

Pulpal diagnosis of teeth treated in PG endodontic clinic	Number of teeth (%)
Previously treated	20 (62)
Symptomatic irreversible pulpitis	8 (25)
Necrotic pulp	2 (6)
Previously initiated	2 (6)

Periapical diagnosis of teeth treated in pg endodontic clinic	Number of teeth (%)
Symptomatic apical periodontitis	23 (72)
Normal apical tissues	6 (19)
Chronic apical abscess	2 (6)
Asymptomatic apical periodontitis	1 (3)
Initiation of endodontic treatment *n* (%)	20 (57)
Completion of endodontic treatment *n* (%)	18 (51)
Reported endodontic complications *n* (%)	3 (14)
Prescription of medication n (%)	6 (17)
Nonsteroidal anti‐inflammatory drug (NSAID)	5 (83)
Chlorhexidine gluconate 0.12% oral rinse	3 (50)
Endodontic non‐compliance^a^ *n*(%)	5 (15)

*Note*: *n* (%): number of patients (percentages). Total number of subjects = 35.

^a^ Endodontic non‐compliance: Patients with whom the endodontic therapy was planned and/or initiated but the patient did not comply with the planned treatment and did not attend the scheduled treatment appointment/s.

### 3.3 Orofacial pain diagnosis, treatment plan, outcomes

**Figure 2:**
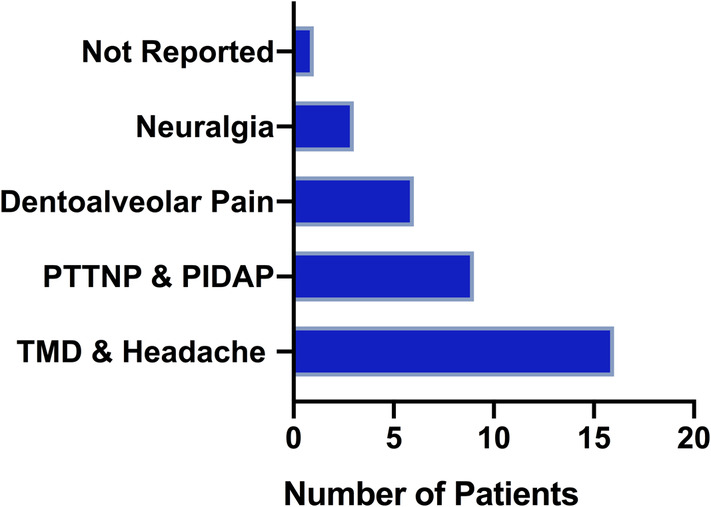
Initial orofacial pain diagnosis. *Temporomandibular disorder* (*TMD*) *and headache*: 15 patients were diagnosed with TMD and 1 patient was diagnosed solely with headache. *Painful traumatic trigeminal neuropathic pain* (*PTTNP*) *and persistent idiopathic dentoalveolar pain* (*PIDAP*): Five patients were described to be diagnosed with PTTNP and four patients were described to be diagnosed with PIDAP. Among those nine patients, four of them were also diagnosed with TMD. *Dentoalveolar pain*: Six patients were diagnosed with pain due to dental or surrounding tissues such as bone, periodontal ligament. *Neuralgia*: Three patients were diagnosed with different types of neuralgia, one of them being diagnosed with trigeminal neuralgia. Numbers show patients

Eighty‐three percent (29/35) of the study subjects were diagnosed with a painful condition of non‐odontogenic origin by the orofacial pain specialist. Differential diagnoses of the non‐odontogenic pain conditions are listed in Figure 2. The majority of patients were diagnosed with TMD, of either joint or muscular origin (19/35, 54%).

In 26% (9/35) of patients there was indication that pain persisted after dental intervention. In seven out of nine, pain persisted after endodontic treatment. In two of the nine, pain persisted after tooth extraction. In the reviewed dental records, varying nomenclatures, such as trigeminal neuropathic pain, were used to describe pain that persisted after dental intervention with accompanying neuropathic pain characteristics (e.g., reports of burning pain) and/or somatosensory changes (e.g., hyperalgesia or allodynia to light touch with a cotton swab to the adjacent buccal mucosa). Out of these nine patients, four were diagnosed with both trigeminal neuropathic pain and TMD, showing that multiple orofacial pain conditions can occur concurrently.

Further, when assessing these nine patients, there was clear evidence that for four of the patients, pain either began or changed its character after the dental intervention of either root canal treatment or tooth extraction. Therefore, in our study, according the current orofacial pain classifications, we described the diagnosis for these patients as PTTNP. For the remaining five patients, because there was not sufficient information from the charts that show dental intervention was the causative trauma, we described the diagnosis for these patients as PIDAP.

Once the patient received a diagnosis from the orofacial pain specialist, different combinations of treatment modalities were prescribed (Table 3). Physical therapy (PT) and pharmacological management were the most frequently prescribed treatments (PT = 16/35 patients, Pharmacological management = 18/35 patients). Other treatments are listed in Table 3. In addition to the described treatment plans, 27 (77%) of the patients were referred by the orofacial pain specialist to different clinicians: physical therapists, dentists, and neurologists.

**Table 3:** Orofacial pain related characteristics

Location of pain *n* (%)	
Maxilla	25 (71)
Mandible	10 (29)
Treatment planned *n* (%)	
Physical therapy	16 (46)
Occlusal guard	8 (23)
Injection therapy	6 (17)
Trigger point injection	5 (83)
Corticosteroid injection	1 (17)
Pharmacological management *n* (%)	18 (51)
Muscle relaxants	8 (44)
Tricyclic antidepressants	5 (28)
Anti‐epileptics	4 (22)
Nonsteroidal anti‐inflammatory drug (NSAID)	3 (17)
Other	2 (11)
Referral to other specialists *n* (%)	27 (77)
Physical therapy	16 (59)
Endodontist	5 (19)
General dentist	4 (15)
Oral surgeon/medicine	4 (15)
Neurologist	4 (15)
Other	2 (7)
Prevalence of pain resolution after OFP treatments *n* (%)	2 (6)
Prevalence of pain reduction after OFP treatments *n* (%)	18 (51)
No reported indication of pain resolution/reduction *n* (%)	15 (43)
Orofacial pain non‐compliance^a^ *n* (%)	23 (66)

*Note*: *n* (%): number of patients (percentages). Total number of subjects = 35.

Abbreviation: OFP, orofacial pain.

^a^ Orofacial pain non‐compliance: Patients that did not attend scheduled follow‐up visits to the OFPC and patients that reported not taking the medications as prescribed in their treatment plan were considered to be non‐compliant in this study.

Complete pain resolution was noted in the charts of only two patients (2/35, 6%), whereas some degree of pain reduction was reported in 51% of all cases (18/35) (Table 3). It is important to note however, that in 43% (15/35) of charts, no information was available regarding pain resolution/reduction.

### 3.4 Treatment timelines and patient compliance

**Figure 3:**
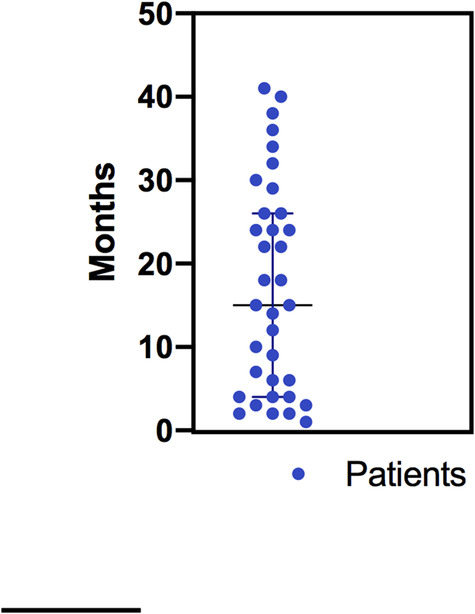
Total duration of care for each patient for diagnosis and treatment including the wait times between appointments. Each dot represents one patient

The total duration of care for treatment of the chief complaint was on average 17 months (Figure 3). This timespan included wait times between appointments. Findings show that 60% (21/35) of patients referred to OFPC presented for at least one unscheduled emergency dental visit due to severity and continuity of pain (Table 1).

With regard to patient compliance, 85% of patients that were presented with an endodontic treatment plan attended the appointments necessary for completion of the root canal therapy (Table 2). Interestingly, compliance was considerably lower—34%—for the treatment prescribed for orofacial pain management (Table 3). Patients that did not attend scheduled follow‐up visits to the OFPC and patients that reported not taking the medications as prescribed in their treatment plan were considered to be non‐compliant in this study.

## 4 DISCUSSION

Determining the correct diagnosis is challenging for both for the clinicians and patients with persistent orofacial pain. Due to the complexity and potential synchronous nature of the factors that contribute to orofacial pain, careful patient examination by a multidisciplinary team is often essential. In this study, all patients were evaluated by at least two types of dental specialists: endodontists and orofacial pain specialists. Ultimately, 83% of the study subjects were diagnosed with a chronic painful condition of non‐odontogenic origin.

In addition to employing a multidisciplinary team when attempting to diagnose chronic orofacial pain, it is critical that examinations include a comprehensive evaluation of the patient's medical history, and take possible risk factors into consideration. Patients with known risk factors for pain chronification may have an increased predilection for developing persistent pain after endodontic treatment and/or have an undiagnosed, chronic orofacial pain condition as a true source of their chief complaint (Kehlet et al., 2006; Penlington et al., 2019; Philpott et al., 2019; Polycarpou et al., 2005; Renton, 2020). Psychological disorders are recognized common comorbidities of patients experiencing chronic orofacial pain and persistent pain after seemingly successful root canal treatment (Aggarwal et al., 2010; Fillingim et al., 2020). In line with the existing literature, in the present study, 43% of the subjects in this study reported histories of depression/anxiety. It is also well described that presence of an ongoing chronic pain condition is a risk factor for developing persistent pain after endodontic therapy (Philpott et al., 2019; Polycarpou et al., 2005). In this study 60% of the cohort reported pre‐existing chronic pain disorders.

Another important aspect of diagnosing orofacial pain that presents as dental pain, is a recognition of pain referral patterns. Pain of non‐odontogenic origin may present as dental pain and potential sources of origin include: orofacial migraine, myofascial, cervical, oropharyngeal, or cardiac pain, and widespread fibromyalgia (De Laat, 2020; Kreiner et al., 2020; Penarrocha et al., 2001; Pigg et al., 2021; Renton, 2020). In our study, the majority (54%) of patients diagnosed with non‐odontogenic pain were diagnosed with TMD. Appropriate recognition of referred pain, understanding of pain temporality, and a comprehensive knowledge of signs and symptoms that are typical of acute dental pain may help clinicians avoid inaccurate diagnosis and unnecessary dental treatment of patients presenting with orofacial pain. For example, 37% of patients in this study were not endodontically treated after evaluation by an endodontic specialist, as their pain was suspected to be of non‐odontogenic origin. In more complex situations, non‐odontogenic orofacial pain can occur concurrently with true odontogenic pain. For example, it is possible that patients who have longstanding tooth pain may develop pain in the muscles of mastication from strain associated with the deliberate avoidance of chewing in a painful area, or from stress‐induced clenching and muscle tension, either of which can ultimately lead to a diagnosis of TMD secondary to the dental pain (Ohrbach & Dworkin, 2016; Slade et al., 2016). In our patient cohort, we found that out of nine patients who had persistent pain after endodontic treatments, four were also diagnosed with TMD, showing that multiple orofacial pain conditions can occur concurrently. It is also possible that pre‐existing TMD condition might have been a risk factor or might have been the actual source of the pain and was referred and presented as odontogenic pain.

Patients presenting with non‐odontogenic pain are often not precluded from dental intervention. Inaccurate diagnosis can be one reason for dental intervention but the synchronous nature of non‐odontogenic orofacial pain and true odontogenic pain described above is another potential reason. In the present study, 71% of patients in this study had a history of endodontic treatment in the quadrant of chief complaint when they presented for endodontic consultation. Further, 63% of subjects were treatment planned for endodontic procedures on one or multiple teeth, prior to referral to the orofacial pain specialists. The retrospective nature of this study does not allow for the determination of the accuracy of the diagnosis that preceded and led to the previous endodontic treatments. However the present findings are consistent with reporting from another retrospective study which found out that 44% of non‐odontogenic orofacial pain patients had a history of endodontic treatment or extraction (Linn et al., 2007). Although dental treatment of patient with complex pain presentation cannot always be avoided, multiple endodontic treatments in the same quadrant where patients present with atypical pain should serve as a reminder that irreversible treatments such as root canal therapy and extraction should be rendered with caution (Zakrzewska, 2007).

The second most frequent chronic orofacial pain diagnoses in this study were attributable to pain persisting after a dental intervention: PIDAP and PTTNP. In this retrospective study, it could only be determined for a fraction of these patients (i.e., who were described with the diagnosis of PTTNP) that a dental intervention was the initiating event for the persistent pain. Most of the patients in this retrospective study reported pain prior to endodontic treatment. However, even though there were reports of pre‐operative pain, it is also possible that the root canal treatment itself may have created an injury that contributed to the persistence of pain (Nixdorf et al., 2016). When we reviewed electronic dental records of these subjects, the orofacial pain specialists noted reports of somatosensory changes (e.g., hyperalgesia to light touch with a cotton swab) accompanying the described pain, for patients with these diagnoses. Therefore, somatosensory testing may be a useful addition to the diagnostic examination process when persistent pain after dental intervention, is suspected (Baad‐Hansen et al., 2013; Benoliel et al., 2012, 2016). For patients diagnosed with PIDAP or PTTNP, systemic pharmacological management was the most common treatment modality (Benoliel & Eliav, 2012). Pain reduction in these patients can be difficult to achieve, and successful management requires finding a proper medication protocol that often must be followed long‐term (Bennett, 2004).

In our study, responses to the orofacial pain treatments varied widely, and in 43% of the charts reviewed there was no documentation of pain relief. One of the reasons for this could be due to low rates of patient return for scheduled follow‐up appointments, making detailed evaluation of treatment outcomes difficult.

The total duration of care for diagnosis and treatment of patients diagnosed with pain of non‐odontogenic origin was, on average, 17 months, including wait times between appointments. Time and financial resource intensiveness may help explain the low patient compliance rate for orofacial pain treatment (34%) in this study. This compliance rate is comparably lower than previous reporting, ranging from 42% to 93% (Riley et al., 1999). Despite this finding, it is clear that the patient profile in this study is not reflective of a generally non‐compliant patient population: 85% of patients presented with an endodontic treatment plan attended the appointments necessary for completion of the root canal therapy. This may be due to the fact that endodontic treatment is perceived to be more definitive in nature than treatment for chronic, orofacial pain. Furthermore, scheduled treatment visits without a clear end‐point and the possible use of medications with significant side effects may negatively impact patient compliance of these treatment plans.

Due to the retrospective nature of this study there are inherent limitations. This is exemplified by our inability to confirm the accuracy of diagnoses and difficulty evaluating pain resolution as a treatment outcome. Additionally, our patient pool was limited, as it was confined to patients treated within a single academic institution. Despite the outlined limitations, our study confirms that the differentiation of non‐odontogenic and odontogenic pain can be challenging. Furthermore, this study highlights that a definitive diagnosis is often only made following assessment from a number of specialists. The involvement of secondary and tertiary referrals makes the process protracted and potentially financially burdensome (Devine et al., 2017; Lang et al., 2016; Linn et al., 2007). As a result of our findings we suggest that when pain of non‐odontogenic nature is suspected, clinicians: (1) Be knowledgeable about temporomandibular joint and muscle disorders, and somatosensory examination when non‐odontogenic pain is suspected. (2) Choose the least invasive treatment modality to rule out continued endodontic pathology. For example, endodontic re‐treatment is relatively non‐invasive, while endodontic surgery can produce additional injury and potentially contribute to pain chronification. (3) When referring patients for orofacial pain evaluation and treatment, it may be a helpful patient management practice to pre‐emptively set patients expectations with regard to time, financial resources and treatment plans.

## 5 CONCLUSIONS

It should be recognized that patients with known risk factors for pain chronification may have an increased predilection for developing persistent pain after endodontic treatment and/or have an undiagnosed, chronic orofacial pain condition as a true source of their chief complaint. A multidisciplinary team is essential for identifying and managing chronic orofacial pain: often a definitive diagnosis is only made following assessments from a number of specialists, involving secondary and tertiary referrals.

## CONFLICT OF INTEREST

The authors declare no conflicts of interest.

## AUTHOR CONTRIBUTIONS

Ozge Erdogan and Lorel L. Burns designed and conducted the study, performed data analysis, and wrote the manuscript. Austin Ramsey performed data collection and edited the manuscript. James M. Uyanik contributed to study design, data collection, analysis, and manuscript editing. Jennifer L. Gibbs contributed to data analysis revised the manuscript. All authors approved the final version of the manuscript.

## Data Availability

The data that support the findings of this study are available from the corresponding author upon reasonable request.

## References

[cre2497-bib-0001] Aggarwal, V. R. , Macfarlane, G. J. , Farragher, T. M. , & McBeth, J. (2010). Risk factors for onset of chronic oro‐facial pain—Results of the North Cheshire oro‐facial pain prospective population study. Pain, 149, 354–359. 20304556 10.1016/j.pain.2010.02.040 PMC2877804

[cre2497-bib-0002] Aggarwal, V. R. , McBeth, J. , Zakrzewska, J. M. , & Macfarlane, G. J. (2008). Unexplained orofacial pain—Is an early diagnosis possible? British Dental Journal, 205, E6 discussion 140‐1, E6; discussion 141. 18596820 10.1038/sj.bdj.2008.585

[cre2497-bib-0003] Baad‐Hansen, L. , Pigg, M. , Ivanovic, S. E. , Faris, H. , List, T. , Drangsholt, M. , & Svensson, P. (2013). Chairside intraoral qualitative somatosensory testing: Reliability and comparison between patients with atypical odontalgia and healthy controls. Journal of Orofacial Pain, 27, 165–170. 23630688 10.11607/jop.1062

[cre2497-bib-0004] Beecroft, E. V. , Durham, J. , & Thomson, P. (2013). Retrospective examination of the healthcare 'journey' of chronic orofacial pain patients referred to oral and maxillofacial surgery. British Dental Journal, 214, E12. 23470414 10.1038/sj.bdj.2013.221

[cre2497-bib-0005] Bennett, G. J. (2004). Neuropathic pain in the orofacial region: Clinical and research challenges. Journal of Orofacial Pain, 18, 281–286. 15636009

[cre2497-bib-0006] Benoliel, R. , Birman, N. , Eliav, E. , & Sharav, Y. (2008). The International Classification of Headache Disorders: Accurate diagnosis of orofacial pain? Cephalalgia, 28, 752–762. 18498396 10.1111/j.1468-2982.2008.01586.x

[cre2497-bib-0007] Benoliel, R. , & Eliav, E. (2012). Neuropathic orofacial pain. The Alpha Omegan, 105, 66–74. 23589946

[cre2497-bib-0008] Benoliel, R. , Teich, S. , & Eliav, E. (2016). Painful traumatic trigeminal neuropathy. Oral and Maxillofacial Surgery Clinics of North America, 28, 371–380. 27475512 10.1016/j.coms.2016.03.002

[cre2497-bib-0009] Benoliel, R. , Zadik, Y. , Eliav, E. , & Sharav, Y. (2012). Peripheral painful traumatic trigeminal neuropathy: Clinical features in 91 cases and proposal of novel diagnostic criteria. Journal of Orofacial Pain, 26, 49–58. 22292140

[cre2497-bib-0010] De Laat, A. (2020). Differential diagnosis of toothache to prevent erroneous and unnecessary dental treatment. Journal of Oral Rehabilitation, 47, 775–781. 32061108 10.1111/joor.12946

[cre2497-bib-0011] Devine, M. , Hirani, M. , Durham, J. , Nixdorf, D. R. , & Renton, T. (2018). Identifying criteria for diagnosis of post‐traumatic pain and altered sensation of the maxillary and mandibular branches of the trigeminal nerve: A systematic review. Oral Surgery, Oral Medicine, Oral Pathology, and Oral Radiology, 125, 526–540. 10.1016/j.oooo.2017.12.020 29426749

[cre2497-bib-0012] Devine, M. , Modgill, O. , & Renton, T. (2017). Mandibular division trigeminal nerve injuries following primary endodontic treatment. A case series. Australian Endodontic Journal, 43, 56–65. 28685976 10.1111/aej.12209

[cre2497-bib-0013] Dieb, W. , Moreau, N. , Chemla, I. , Descroix, V. , & Boucher, Y. (2017). Neuropathic pain in the orofacial region: The role of pain history. A retrospective study. Journal of Stomatology, Oral and Maxillofacial Surgery, 118, 147–150. 10.1016/j.jormas.2017.03.004 28365394

[cre2497-bib-0014] Fillingim, R. B. , Ohrbach, R. , Greenspan, J. D. , Sanders, A. , Rathnayaka, N. , Maixner, W. , & Slade, G. (2020). Associations of psychologic factors with multiple chronic overlapping pain conditions. Journal of Oral & Facial Pain and Headache, 34, s85–s100. 32975543 10.11607/ofph.2584 PMC10165716

[cre2497-bib-0015] Harris, P. A. , Taylor, R. , Thielke, R. , Payne, J. , Gonzalez, N. , & Conde, J. G. (2009). Research electronic data capture (REDCap)—A metadata‐driven methodology and workflow process for providing translational research informatics support. Journal of Biomedical Informatics, 42, 377–381. 18929686 10.1016/j.jbi.2008.08.010 PMC2700030

[cre2497-bib-0037] ICOP . (2020). International Classification of Orofacial Pain, 1st edition. Cephalalgia, 40(2), 129–221. 10.1177/0333102419893823 32103673

[cre2497-bib-0016] Kehlet, H. , Jensen, T. S. , & Woolf, C. J. (2006). Persistent postsurgical pain: Risk factors and prevention. Lancet, 367, 1618–1625. 16698416 10.1016/S0140-6736(06)68700-X

[cre2497-bib-0017] Kreiner, M. , Okeson, J. , Tanco, V. , Waldenstrom, A. , & Isberg, A. (2020). Orofacial pain and toothache as the sole symptom of an acute myocardial infarction entails a major risk of misdiagnosis and death. Journal of Oral & Facial Pain and Headache, 34, 53–60. 31465031 10.11607/ofph.2480

[cre2497-bib-0018] Lang, M. , Selvadurai, T. , & Zakrzewska, J. M. (2016). Referrals to a facial pain service. British Dental Journal, 220, 345–348. 27056518 10.1038/sj.bdj.2016.262

[cre2497-bib-0019] Linn, J. , Trantor, I. , Teo, N. , Thanigaivel, R. , & Goss, A. N. (2007). The differential diagnosis of toothache from other orofacial pains in clinical practice. Australian Dental Journal, 52, S100–S104. 17546865 10.1111/j.1834-7819.2007.tb00518.x

[cre2497-bib-0020] Lipton, J. A. , Ship, J. A. , & Larach‐Robinson, D. (1993). Estimated prevalence and distribution of reported orofacial pain in the United States. Journal of the American Dental Association (1939), 124, 115–121. 10.14219/jada.archive.1993.0200 8409001

[cre2497-bib-0021] Macrae, W. A. (2008). Chronic post‐surgical pain: 10 years on. British Journal of Anaesthesia, 101, 77–86. 18434337 10.1093/bja/aen099

[cre2497-bib-0022] Nixdorf, D. R. , Law, A. S. , Lindquist, K. , Reams, G. J. , Cole, E. , Kanter, K. , Nguyen, R. H. N. , Harris, D. R. , & National Dental PBRN Collaborative Group . (2016). Frequency, impact, and predictors of persistent pain after root canal treatment: A national dental PBRN study. Pain, 157, 159–165. 26335907 10.1097/j.pain.0000000000000343 PMC4684798

[cre2497-bib-0023] Nixdorf, D. R. , Moana‐Filho, E. J. , Law, A. S. , McGuire, L. A. , Hodges, J. S. , & John, M. T. (2010). Frequency of nonodontogenic pain after endodontic therapy: A systematic review and meta‐analysis. Journal of Endodontia, 36, 1494–1498. 10.1016/j.joen.2010.06.020 PMC2941431 20728716

[cre2497-bib-0024] Ohrbach, R. , & Dworkin, S. F. (2016). The evolution of TMD diagnosis: Past, present, future. Journal of Dental Research, 95, 1093–1101. 27313164 10.1177/0022034516653922 PMC5004241

[cre2497-bib-0025] Penarrocha, M. , Bandres, A. , Penarrocha, M. A. , & Bagan, J. V. (2001). Relationship between oral surgical and endodontic procedures and episodic cluster headache. Oral Surgery, Oral Medicine, Oral Pathology, Oral Radiology, and Endodontics, 92, 499–502. 10.1067/moe.2001.116153 11709684

[cre2497-bib-0026] Penlington, C. , Araujo‐Soares, V. , & Durham, J. (2019). Predicting persistent orofacial pain: The role of illness perceptions, anxiety, and depression. JDR Clinical and Translational Research, 5, 40–49. 31063437 10.1177/2380084419846447

[cre2497-bib-0027] Philpott, R. , Gulabivala, K. , Leeson, R. , & Ng, Y. L. (2019). Prevalence, predictive factors and clinical course of persistent pain associated with teeth displaying periapical healing following nonsurgical root canal treatment: A prospective study. International Endodontic Journal, 52, 407–415. 30332512 10.1111/iej.13029

[cre2497-bib-0028] Pigg, M. , Nixdorf, D. R. , Law, A. S. , Renton, T. , Sharav, Y. , Baad‐Hansen, L. , & List, T. (2021). New international classification of orofacial pain: What is in it for endodontists? Journal of Endodontia, 47, 345–357. 10.1016/j.joen.2020.12.002 33340605

[cre2497-bib-0029] Polycarpou, N. , Ng, Y. L. , Canavan, D. , Moles, D. R. , & Gulabivala, K. (2005). Prevalence of persistent pain after endodontic treatment and factors affecting its occurrence in cases with complete radiographic healing. International Endodontic Journal, 38, 169–178. 15743420 10.1111/j.1365-2591.2004.00923.x

[cre2497-bib-0030] Renton, T. (2020). Tooth‐related pain or not? Headache, 60, 235–246. 31675112 10.1111/head.13689

[cre2497-bib-0031] Riley, J. L., III , Robinson, M. E. , Wise, E. A. , Campbell, L. C. , Kashikar‐Zuck, S. , & Gremillion, H. A. (1999). Predicting treatment compliance following facial pain evaluation. Cranio, 17, 9–16. 10425925 10.1080/08869634.1999.11746072

[cre2497-bib-0032] Slade, G. D. , Ohrbach, R. , Greenspan, J. D. , Fillingim, R. B. , Bair, E. , Sanders, A. E. , Dubner, R. , Diatchenko, L. , Meloto, C. B. , Smith, S. , & Maixner, W. (2016). Painful temporomandibular disorder: Decade of discovery from OPPERA studies. Journal of Dental Research, 95, 1084–1092. 27339423 10.1177/0022034516653743 PMC5004239

[cre2497-bib-0033] Vena, D. A. , Collie, D. , Wu, H. , Gibbs, J. L. , Broder, H. L. , Curro, F. A. , Thompson, V. P. , Craig, R. G. , & PEARL Network Group . (2014). Prevalence of persistent pain 3 to 5 years post primary root canal therapy and its impact on oral health‐related quality of life: PEARL network findings. Journal of Endodontia, 40, 1917–1921. 10.1016/j.joen.2014.07.026 25220076

[cre2497-bib-0034] Wright, E. F. (2000). Referred craniofacial pain patterns in patients with temporomandibular disorder. Journal of the American Dental Association (1939), 131, 1307–1315. 10986831 10.14219/jada.archive.2000.0384

[cre2497-bib-0035] Wright, E. F. , & Gullickson, D. C. (1996). Identifying acute pulpalgia as a factor in TMD pain. Journal of the American Dental Association (1939), 127, 773–780. 8708279 10.14219/jada.archive.1996.0313

[cre2497-bib-0036] Zakrzewska, J. M. (2007). Diagnosis and management of non‐dental orofacial pain. Dental Update, 34(134–6), 8–9. 10.12968/denu.2007.34.3.134 17506453

